# Pathological Changes and CYP1A1 Expression as Biomarkers of Pollution in Sarpa Salpa and Diplodus Sargus

**DOI:** 10.3390/ani14213160

**Published:** 2024-11-04

**Authors:** Maria Dimatteo, Evaristo Di Napoli, Orlando Paciello, Ilaria d’Aquino, Doriana Iaccarino, Marianna D’amore, Mariangela Guida, Luciana Cozzolino, Francesco Paolo Serpe, Giovanna Fusco, Esterina De Carlo, Barbara degli Uberti

**Affiliations:** 1 Istituto Zooprofilattico Sperimentale del Mezzogiorno, 80035 Portici, Italy; doriana.iaccarino@izsmportici.it (D.I.); marianna.damore@izsmportici.it (M.D.); mariangela.guida@izsmportici.it (M.G.); francesco.serpe@izsmportici.it (F.P.S.); giovanna.fusco@izsmportici.it (G.F.); esterina.decarlo@izsmportici.it (E.D.C.); barbara.degliuberti@izsmportici.it (B.d.U.); 2 Unit of Pathology, Department of Veterinary Medicine and Animal Production, University of Naples Federico II, 80137 Naples, Italy; paciello@unina.it (O.P.); ilaria.daquino@unina.it (I.d.); lucianacozzolinovet@gmail.com (L.C.)

**Keywords:** pollution, teleost fishes, lead, non-dioxin-like polychlorinated biphenyls, environmental contaminants, CYP1A1

## Abstract

In a marine ecosystem, the most sensitive organisms to environmental changes, mainly to anthropic pressures, are fishes and invertebrates. Therefore, they are considered the ideal targets to indirectly evaluate the health of an entire ecosystem. Teleost fishes, particularly those that occupy the highest trophic levels, can accumulate toxic substances through their diet. In this study, we used two fish species with sedentary behavior and trophic habits, *Diplodus sargus* and *Sarpa salpa*, caught in two areas at different anthropic pressures divided into the Gulf of Naples (Na) and the Gulf of Salerno (Sa). This study aimed to correlate the pathological alterations in target organs in both species with known concentrations of polychlorinated biphenyls (PCBs) and heavy metals (lead and cadmium) to the expression of CYP1A1. Histological examination took into consideration circulatory disorders, increase in melanomacrophages (MMs) number, inflammation in kidney and hepatopancreas and gonadal stage maturation. Next, the pathological and morphological changes found were compared to immunohistochemical expression of CYP1A1 in the same samples. Chemical analysis of PCBs, based on 28, 52, 101, 138, 153, and 180 congeners, and heavy metals, were performed on hepatopancreas and muscle samples. Higher median values of PCBs concentration were detected in both species in the Salerno area (8.1 ng/g in *Diplodus sargus* muscles and 51.1 ng/g in *Sarpa salpa* hepatopancreas, respectively), although the values were consistently below the legal limits. No critical values were found for lead and cadmium. Therefore, we hypothesized that CYP1A1 and pathological alterations were more expressed in fish from Salerno area. The pathological changes showed a statistically significant difference in inflammation of the kidneys (*p* < 0.0001) between *S. salpa* of both Gulfs. In addition, we found a statistically significant difference in the assessment of the increase in MMs/MMCs (*p* = 0.0384) and circulation disorders (*p* = 0.0325) of hepatopancreas in *D. sargus* of both Gulfs. As not all the variables considered showed statistical significance, the analysis of the results does not fully support the correlation between the highest levels of contaminants found in the Salerno area and the expression of CYP1A1. Our data could be a starting point for future studies to better correlate the role of CYP1A1 to pollutants, considering this is the first study involving two of the most common species in the Mediterranean Sea. Thus, future studies could include other species to improve and increase records.

## Simple Summary

In a marine ecosystem, the organisms most sensitive to the effects of environmental pollution are fish and invertebrates. Therefore, they are considered ideal targets to indirectly assess the health of an entire ecosystem. Teleost fish, especially those occupying the highest trophic levels, can accumulate toxic substances through their diet. In this study, we used two fish species with sedentary behavior and trophic habits, *Diplodus sargus* and *Sarpa salpa*, caught in two areas with different anthropic pressures divided into the Gulf of Naples (Na) and the Gulf of Salerno (Sa). This study aimed to correlate the pathological alterations in target organs in both species with known concentrations of polychlorinated biphenyls (PCBs) and heavy metals (lead and cadmium) to the expression of CYP1A1 to assess the health of a whole ecosystem.

## 1. Introduction

Pollution is one of the unsolved issues that impacts all the different ecosystems in the world, including aquatic species. In our modern healthcare system, the “one health” approach has introduced a new concept, different ecosystems are strictly interconnected and dependent on each other. Consequently, the integration of different disciplines and sectors is essential to prevent any threats to health [1]. Thus, the effects of pollution on the environment can be studied by monitoring ecological variations that include organisms and biological parameters. Biomonitoring marine environments could potentially prevent disease in aquatic animals and humans [2]. Several international programs are carried out on different fish species, such as Mahi mahi (*Coryphaena hippurus*), Red drum (*Sciaenops ocellatus*) [3], Sea bass (*Dicentrarchus labrax*) [4], *Zeus faber*, and *Lepidopus caudatus* [5], including freshwater species like Fathead minnow (*Pimephales promelas*) [6], to assess their diversity and biomass [7]. Therefore, to investigate the damage to biota from pollution, it is useful to measure and monitor biological indicators, known as biomarkers, of the involved species and their habitats [8]. In teleosts, it was observed that some enzymes may act as intermediate receptors in the activation of cytochrome P450-dependent monooxygenases (CYP1A). Indeed, it was described that 7-ethoxyresorufin-O-deethylase (EROD) in the liver and gills can induce the activation of CYP1A due to environmental contaminants’ exposure to planarhalogenated/polycyclic aromatic hydrocarbons (PHH/PAH) [9]. The CYP1A enzyme includes two different isoforms, CYP1A1 and CYP1A2, in mammals and teleost fishes, as reported in rainbow trout [10].

Its role is to convert lipophilic xenobiotics by monooxygenation to more water-soluble metabolites [11]. This gene also triggers after the aryl hydrocarbon hydroxylase (AHH) receptor binds to pollutants such as polychlorinated biphenyls (PCBs), polyaromatic hydrocarbons (PAHs), dioxins, furans, and heavy metals [12,13,14]. In fish, the effects of these environmental contaminants, in particular PCBs and heavy metals, are related to reproductive and development disorders [15,16], liver damage, and neurobehavioral effects. Furthermore, in mammals, they are also associated with teratogenicity [17]. Lead (Pb) and cadmium (Cd) are the most widespread heavy metals in marine eco-systems. Indeed, fishes exposed to high lead levels experience synaptic damage, neurodegenerative disorders, and cognitive function damage. Moreover, long-term exposure to lead is reported to act as a suppressor of the immune system [18]. Equally, in humans, long-term exposure to heavy metals is reported to have the most significant effect in children. Indeed, due to the higher gastrointestinal uptake compared to adults, as well as their incomplete blood–brain barrier [19], children are more susceptible to heavy metal effects.

According to the European Food Safety Authority (EFSA), detectable levels of PCBs contamination have been found in multiple fishes and fishery products as well as in products of animal origin [20]. Indeed, PCBs are recognized as Persistent Organic Pollutants (POPs), resulting in high persistence in the marine environment, due to their excessive use in the past years [21]. They also represent a serious poisoning contaminant for human and environmental health [13,22]. The present study aimed to investigate the interspecies variability to contaminant exposure among two different fish species, *Sarpa salpa* and *Diplodus sargus*, characterized by sedentary behavior, different trophic habits, season, and reproduction. In particular, we evaluated the expression of CYP1A1 in the hepatopancreas and kidney of the species under investigation, which were collected from two different areas with known concentrations of PCBs and heavy metals.

## 2. Materials and Methods

### 2.1. Sampling and Fish Examination

The collection areas were located along the Tyrrhenian coastline in the Campania region. The first area extended from Massa Lubrense to Vico Equense in the Gulf of Naples (Na), and the second one from Reccomone Bay to the rock of Isca, within the Marine Protected Area of Punta Campanella in the Gulf of Salerno (Sa). The areas under investigation are already reported by Santoro and colleagues (2020) [23]. The collection took place between February and December 2017 for a total of 242 fishes, including 120 *Diplodus sargus* (64 and 56 from Na and Sa) and 122 *Sarpa salpa* (60 and 62 from Na and Sa) caught by gillnet fisheries at 15 to 30 m depths. The fishes involved in this study were collected during standard routine fishery procedures according to Italian law DL16/92 and European directive 2010/63/EU; therefore, no specific permit was required. Each animal primarily underwent a macroscopic examination and parasitological study, and environmental data concerning 2013–2018 were obtained from the Regional Agency for Environmental Protection of Campania (ARPAC) website [24].

### 2.2. Chemical Analysis

#### 2.2.1. Non-Dioxin-like Polychlorinated Biphenyls (NDL-PCBs)

To determine the 6 congener concentrations of the PCB “indicators” (Commission Regulation UE 2023/915 of 25 April 2023) in the Gulf of Naples, 23 muscle and 7 hepatopancreas samples of *Sarpa salpa* were analyzed. The muscles were pooled in 6 pools. Hepatpancreas samples were pooled in 3 pools. Ten *Diplodus sargus* muscle samples were analyzed and divided into 2 pools, and four hepatopancreas samples were pooled in 1 pool. In the Gulf of Salerno, a total of 8 *Sarpa salpa* muscle samples, pooled in 2 pools, eight hepatopancreas samples, pooled in 2 pools, 8 *Diplodus sargus* muscle samples, pooled in 2 pools, and 8 hepatopancreas samples, pooled in 2 pools, were analyzed. The analyses of PCBs specifically concerned the following congeners: 28, 52, 101, 138, 153, and 180, and the studies were carried out by gas chromatography-mass spectrometry with a high-resolution detector (HRGC/HRMS) (Method 1668C 2010). The sample preparation phase involved, after defrosting and taking 2.0 g of fresh weight for both the muscle and the hepatopancreas, an extraction phase with ethyl ether for 24 h and subsequent addition of 50.0 µL of a standard extraction solution of the 6 13C-labeled NDL-PCB congeners. The extract was then transferred into 100 mL conical bottom flasks; the neck was previously filled with quartz wool and anhydrous sodium sulfate. The sample extract was then dried using a rotary evaporator. The fat obtained was taken up with a mixture of *n*-hexane: dichloromethane (1:1) and subsequently mineralized on Extrelut NT3 previously conditioned with 3 mL of 96% sulfuric acid [25], eluted with 2 fractions of 10 mL of hexane toluene (8:2), and brought to a small volume with the aid of a rotary evaporator. Subsequently, the sample was taken up with 2 mL of hexane and purified using 6 mL Florisil SPE columns conditioned with 2 fractions of 5 mL of hexane and subsequent elution with 2 fractions of 5 mL of dichloromethane. The sample was then collected in a 100 mL flask, reduced to a volume of approximately 0.2 mL, and subsequently transferred into 300 µL vials in which 5 µL of tetradecane was previously added plus approximately 100 µL of *n*-hexane used for washing the flask after transfer to the vials. The samples in the vials were brought to a small volume in a vacuum centrifuge. The samples were then filled with 50 µL of internal standard injection solution. Subsequently, a 1:5 dilution was carried out with nonane. Finally, the samples were injected and read using HRGC-HRMS (DFS) for quantitative analysis.

#### 2.2.2. Heavy Metals

The same samples tested for PCB levels were also tested for the determination of lead and cadmium. They were collected and stored at −20 °C until analyzed. Tissues were thawed and homogenized, and then the aliquots (0.75 ± 0.01 g) were digested in 4.0 mL of 70% nitric acid, 1.5 mL of 30% hydrogen peroxide, and 3.5 mL of ultrapure water for atomic absorption spectroscopy in a microwave digestion system under high pressure and temperature of 190 °C. Digested samples were analyzed for quantitative determination of lead and cadmium by an atomic absorption spectrophotometer equipped with graphite furnace atomizer with Zeeman effect (GF-AAS), in the presence of matrix modifiers (monobasic ammonium phosphate and magnesium nitrate 1% Mg). Standard solutions of lead and cadmium were prepared by diluting multi-elemental standard solutions of 1000 mg L^−1^, and working standard solutions of heavy metals were prepared by diluting stock solutions with ultrapure water. Quantification was performed by external standardization, with correction for recovery percentage. Calibration curves were obtained by analyzing the standard solutions of each metal. Concentrations were expressed as mg kg of wet weight. Analysis was carried out according to [25].

### 2.3. Histopathological Examination

One hundred ninety-nine fishes were analyzed, including one hundred *Diplodus sargus* (40 and 60 from Sa and Na, respectively) and ninety-nine *Sarpa salpa* (45 and 54 from Sa and Na, respectively). From each animal, gonads, hepatopancreas, kidney, and brain were collected. Samples were fixed in 10% buffered formalin and embedded in paraffin. Then, 3–4 µm thick sections were stained with hematoxylin and eosin with a standard protocol (Leica Autostainer XL, Buccinasco (MI) Italy). Histological changes were classified as follows: (1) presence and absence of circulatory disturbance (hemorrhage/hyperemia); (2) number of melanomacrophages (MMs) and melanomacrophage centers (MMCs); (3) inflammation. In particular, some authors investigated the presence of MMs in teleosts in liver and kidney [26,27,28] and correlated their increase in number, volume, and size to stress conditions such as parasites, temperature changes, and pollutants [29]. Due to the absence of a standardized system in the literature for the count of MMs and for the classification of flogosis, our laboratory evaluated the following score system. The number of MMs and MMCs was counted in 10 randomly chosen and not overlapping fields at high power magnification (HPFs, 7.8 area mm^2^) and then the increase in number was scored as follows:

- Score 0 (absent): 1 to 5;
- Score 1 (mild): 6 to 10;
- Score 2 (moderate): 11 to 49;
- Score 3 (severe): >50.

Inflammation was scored as follows: 0 absent, 1 mild (focal distribution in the tissue), 2 moderate (multifocal distribution in the tissue), and 3 severe (diffuse distribution in the tissues). The gonadal stage was histologically evaluated in both species following the method proposed by Shinkafi and colleagues [30]. Both species’ different development phases of oocytes and spermatocytes were associated with a specific gonad maturation stage ranging from I to VI, where I indicated immature, II maturing, III mature, IV ripe and running, V spent, and VI resting.

### 2.4. Immunohistochemistry

Immunohistochemistry (IHC) for anti-CYP1A1 rabbit polyclonal antibody (H-70) (sc-20772—Santa Cruz Biotecnology, Inc., 2145 Delaware Ave, Santa Cruz, CA, USA) was performed on hepatopancreas and kidney samples. First, 3–4 μm thick sections of hepatopancreas and kidney were mounted on positively charged glass slides (Bio-Optica, Milan, Italy) and deparaffined in xylene, and decreasing series of alcohol and peroxidases were blocked with a solution of hydrogen peroxide and methanol (4:1) for 15 min. Antigen retrieval pretreatments were executed using a heat-induced epitope retrieval (HIER) citrate buffer pH 6.0 (Bio-Optica, Milan, Italy) for 20 min at 98 °C. Further, immunohistochemistry was carried out following the protocol suggested by the MACH1 Universal HRP-Polymer Detection Kit (Cat. No: M1U539 G, L10, Bio-Optica, Milan, Italy). Sections were blocked with a protein block (MACH1, Biocare Medical LLC., Concord, CA, USA) for 30 min. Slides were incubated overnight at 4 °C with the primary antibody diluted in phosphate-buffered saline (PBS) (0.01 M PBS, pH 7.2). The primary antibody was a rabbit polyclonal antibody raised against amino acids 246–315, mapping to an internal region of CYP1A1 of human origin at 1:400 dilution. Antibody deposition was visualized using the 3,3′-diaminobenzidine (DAB) chromogen diluted in the DAB substrate buffer; subsequently, the slides were counterstained with hematoxylin. Slides were washed twice (5 min each) in PBS between all incubation steps. In the corresponding negative control sections, the primary antibody was omitted or replaced with a 1:20 dilution of rabbit serum (Code 011-000-120, Jackson Immuno Research, West Grove, PA, USA).

As negative control tissue, we used muscle samples from the same animals [11].

Slides were examined with a standard light microscope and photographed with a Pannoramic 250 Flash III slide scanner. Immunopositivity expression was classified as (1) focal cytoplasmic staining and (2) diffuse cytoplasmic staining.

### 2.5. Statistical Analysis

We used the Mann–Whitney test, a nonparametric test, for statistical analysis. We evaluated the circulatory disturbances, increased melanomacrophages, inflammation, and immunohistochemical expression of CYP1A1 in kidneys and hepatopancreas of Diplodus sargus and Sarpa salpa of both Gulfs. The statistical analysis results were considered statistically significant for *p* < 0.05.

## 3. Results

### 3.1. Chemical

#### 3.1.1. NDL-PCBs

**Graph 1:**
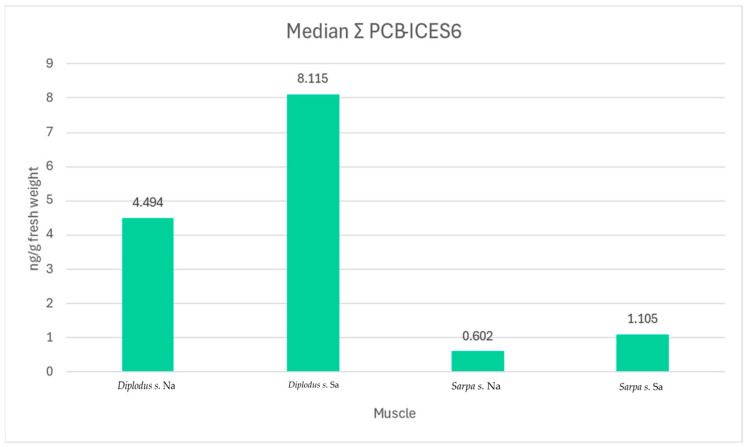
Muscle interspecies comparison of PCBs concentration in the two studied areas.

**Graph 2:**
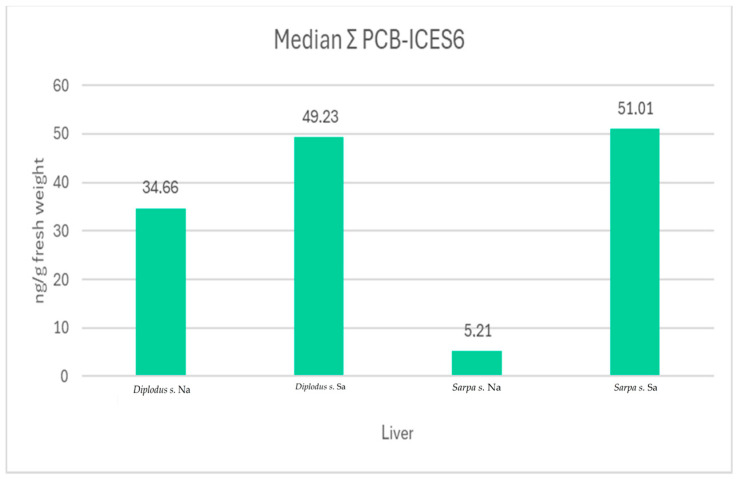
Liver interspecies comparison of PCBs concentration in the two studied areas.

For the Gulf of Naples area, PCB median values of 4.5 and 0.6 ng/g of fresh weight were found for muscle in *Diplodus sargus* and *Sarpa salpa*, respectively; median values of 34.7 and 5.2 ng/g of fresh weight of hepatopancreas were found in *Diplodus sargus* and *Sarpa salpa*, respectively. For the Gulf of Salerno area, median values of 8.1 and 1.1 ng/g of fresh weight were found for the muscle and median values of 49.3 and 51.0 ng/g of fresh weight were found for the hepatopancreas of *Diplodus sargus* and *Sarpa salpa*, respectively (Graph 1 and Graph 2).

#### 3.1.2. Heavy Metals

Among 23 muscle samples, a median lead level of 0.022 mg/kg with a minimum value of “undetectable” (<0.020 mg/kg) and a maximum of 0.224 mg/kg of fresh weight was detected; these findings indicate a level of background contamination that cannot be compressed for the area of the Gulf of Naples. Conversely, in all the samples, cadmium was below the limit of quantification of the method for all samples. Levels of lead are reported in Table 1 and Table 2. No critical values emerged or were above the maximum limit fixed for human consumption.

### 3.2. Histopathological Examination

**Graph 3:**
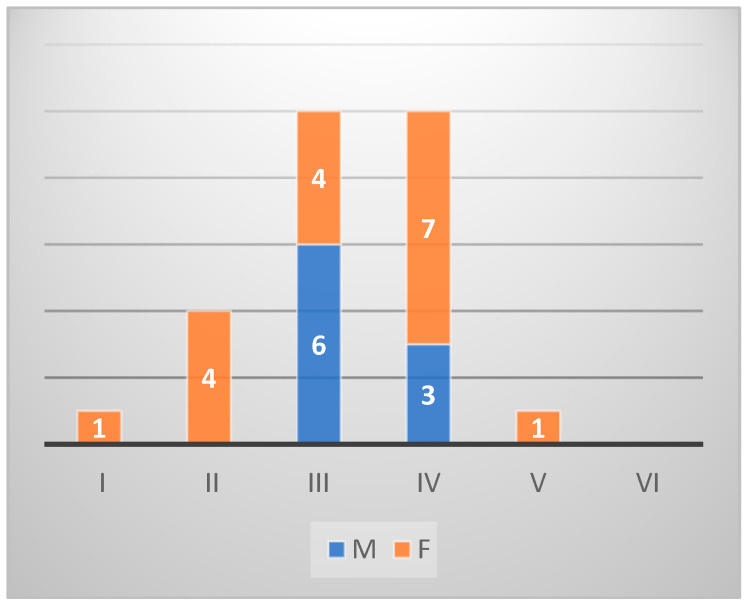
Gonad maturation stage in *Diplodus sargus* from Sa.

*Diplodus sargus* individuals (*n* = 40) from Sa were histologically identified as 15 males and 17 females. In particular, five were classified in an undifferentiated stage, and three were hermaphrodites. Among the female individuals, seven were in stage IV, four in the stage III, four in stage II, one in the stage I, and one in stage V. Among the males, six were in stage III and nine in stage IV (Graph 3).

**Graph 4:**
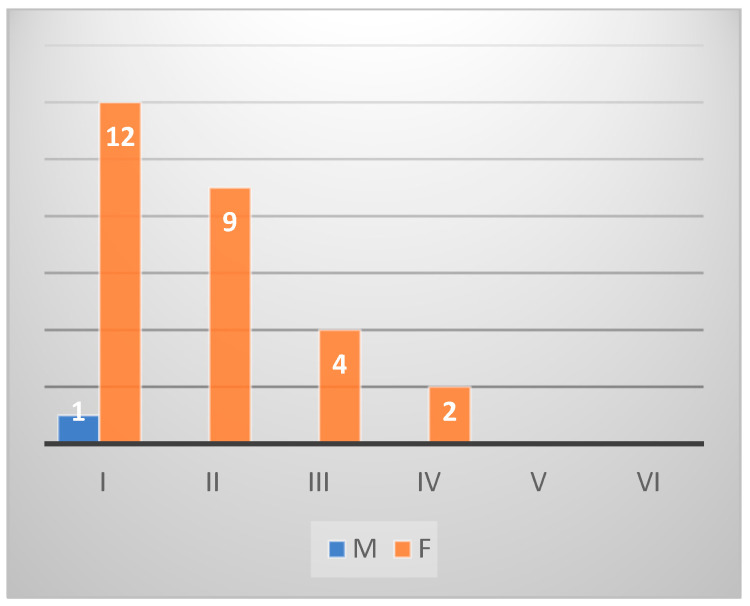
Gonad maturation stage in *Sarpa salpa* from Sa.

**Graph 5:**
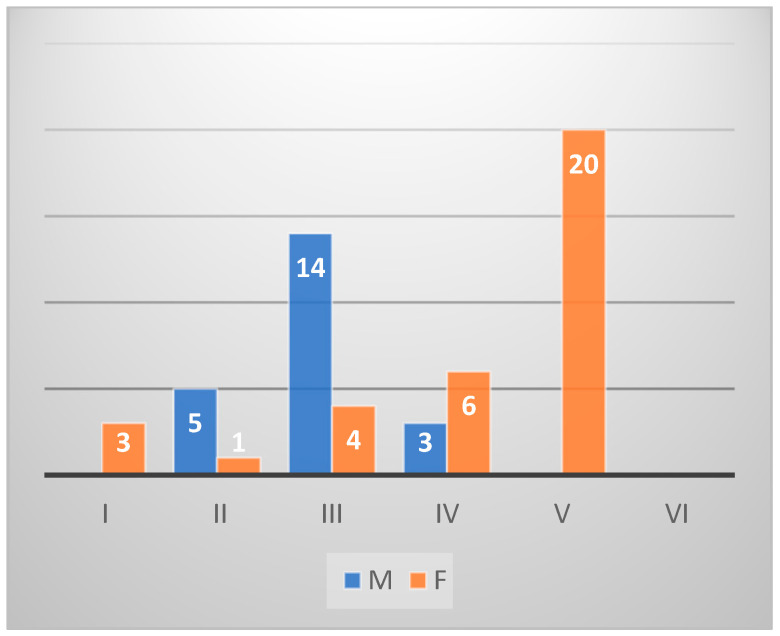
Gonad maturation stage in *Diplodus sargus* from Na.

**Graph 6:**
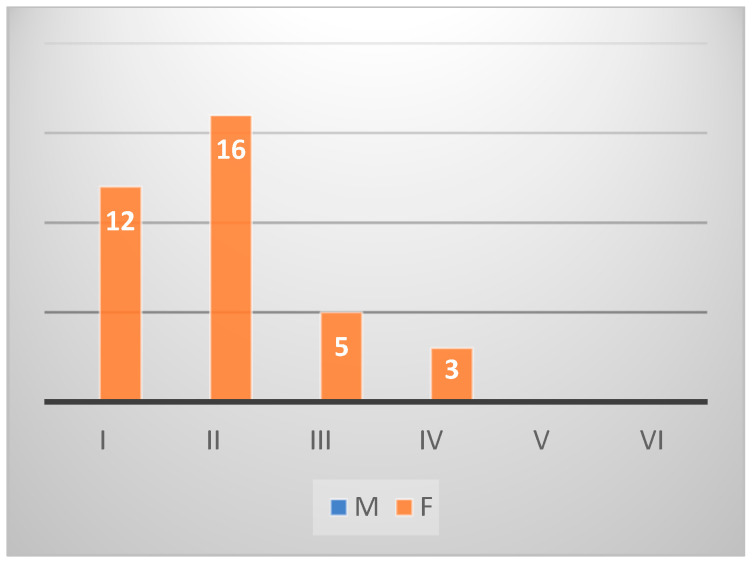
Gonad maturation stage in *Sarpa salpa* from Na.

*Sarpa salpa* fishes (*n* = 45) of the same area counted only one male in stage I, 27 female fishes, 7 hermaphrodites, and 10 undifferentiated. Twelve females were classified in stage I, nine in stage II, four in stage III, and two in stage IV (Graph 4). Of a total of 60 *Diplodus sargus* from Na, 22 were identified as males, 34 as females, and 4 as hermaphrodites. The males were classified as 14 in stage III, 5 in stage II, and 3 in stage IV. Among the females, 20 were in stage V, 6 in stage IV, 4 in stage III, 3 in stage I, and 1 in stage II (Graph 5). In the group of *Sarpa salpa* individuals (*n* = 54) of the same catching area, there were no males, 36 females, 15 undifferentiated phases, and 3 hermaphrodites. The gonad maturation stage established 16 females in stage II, 12 in stage I, 5 in stage III, and 3 in stage IV (Graph 6).

Histological evaluation of hepatopancreas and kidney for the three categories considered is reported in Table 3.

**Figure 1:**
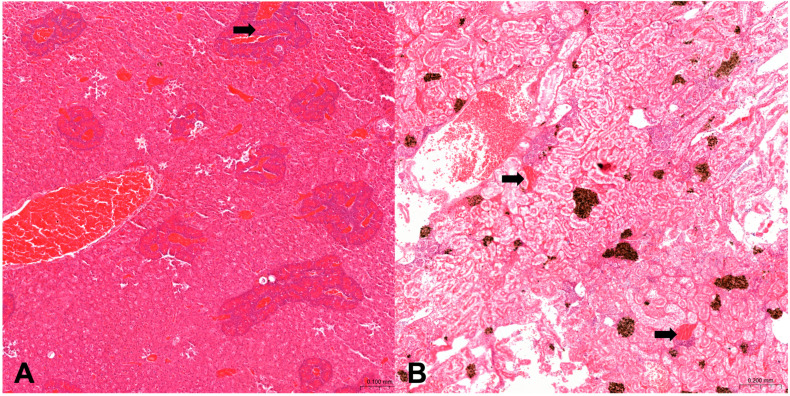
Histological sections of hepatopancreas (**A**) and kidney (**B**) in a *D. sargus* showing congestion and hemorrhage (hematoxylin and eosin, (4×)). (**A**) In liver, normal pancreatic tissue, known as hepatopancreas, invades the branches of the portal veins that appear congested (black arrow). (**B**) Several hemorrhage areas in the interstitiutm between renal tubules (black arrows).

Circulatory disturbances (Figure 1) were observed as follows: in 37.5% (15/40) of both organs of *Diplodus sargus* from Sa, in 21.7% (13/60) of kidneys and 16.7% (10/60) of hepatopancreas of *Diplodus sargus* from Na, 11.1% (5/45) of kidneys and 22.2% (10/45) of hepatopancreas of *Sarpa salpa* from Sa, and 18.5% (10/54) of kidneys and 11.1% (6/54) of hepatopancreas of *Sarpa salpa* from Na.

**Figure 2:**
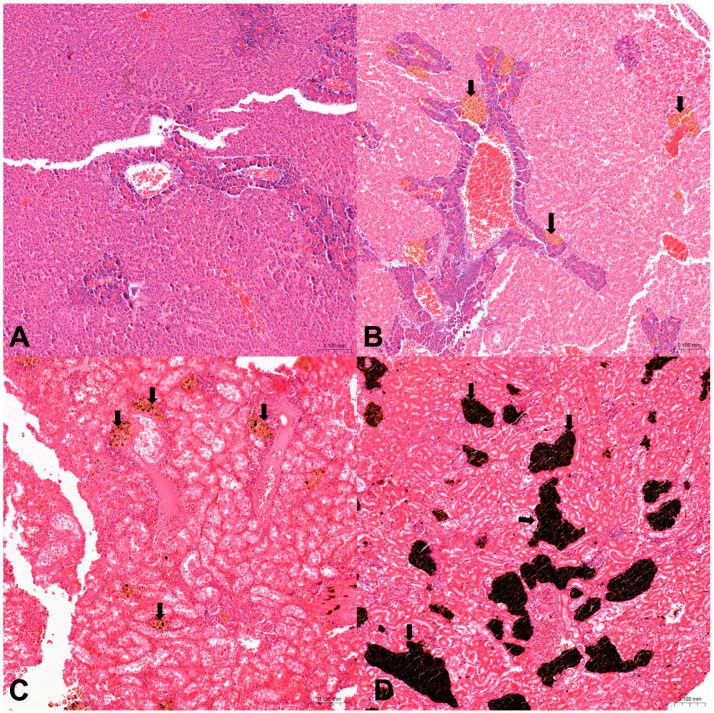
Histological sections of hepatopancreas (**A**,**B**) in *S. salpa* from Sa and kidney in *D. sargus* from Sa (**C**,**D**) (hematoxylin and eosin, (10×)). (**A**) Normal hepatopancreas tissue with no increase in MMs (grade 0). (**B**) Hepatopancreas tissue with increase in number of MMs (grade 2), (black arrows). (**C**) Renal tissue with few MMs (grade 1) and (**D**) with substantial increase in MMs (grade 3), (black arrows).

Regarding the increase in number of MMs and MMCs (Figure 2) the results are summarized in Table 4. In *Diplodus sargus* from SA, 15% of both organs showed an increased number in MMs and MMcs. Among these, 7.5% (3/40) of both organs showed as grade 1, 5% (2/40) of both organs showed as grade 3, and 2.5% (1/40) of both organs showed as grade 2. In *Diplodus sargus* from Na, only 1.7% (1/60) of the hepatopancreas were grade 1, and 8.3% of kidneys resulting in an increase in number of MMs and MMCs were scored as follows: 3.3% (2/60) as grade 3; 3.3% (2/60) as grade 2; and 1.7% (1/60) as grade 1. In *Sarpa salpa* from Sa, 11.1% of hepatopancreas were considered with an increased number in MMS and MMCs and scored as follows: 6.7% (3/45) as grade 2; 2.2% (1/45) as grade 3; and 2.2% (1/45) as grade 1. Of kidneys with an increased number, 6.7% were scored as follows: 2.2% (1/45) as grade 1; 4.4% (2/45) as grade 2.

In *Sarpa salpa* from Na, hepatopancreas showed a 3.70% (2/54) increased number scored as grade 2.

**Figure 3:**
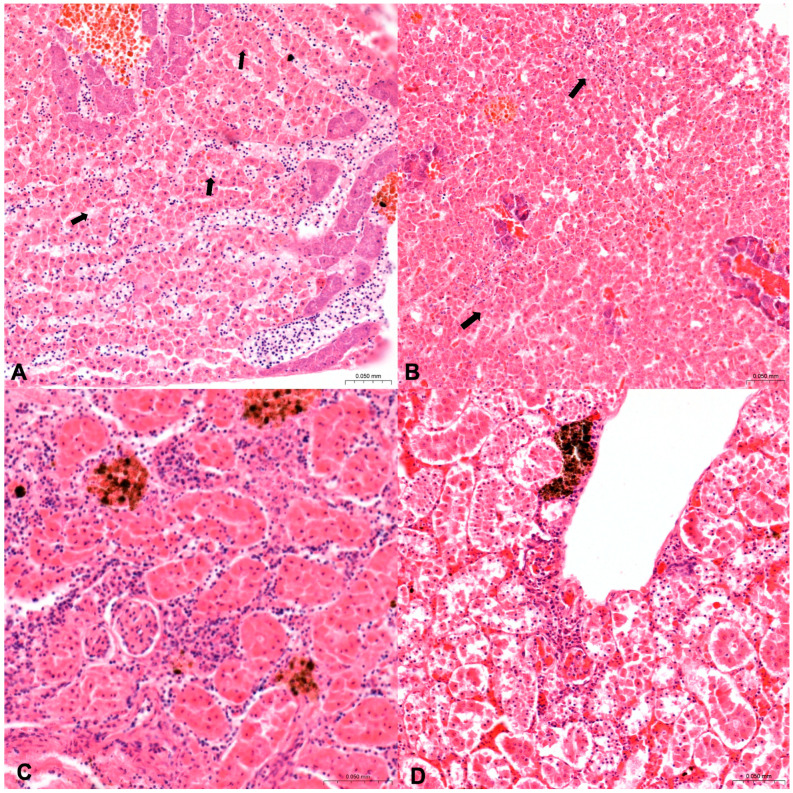
Histological sections of hepatopancreas (**A**,**B**) in *S. salpa* from Na and kidney (**C**,**D**) in *D. sargus* from Na (hematoxylin and eosin, (20×)). (**A**) Several inflammatory infiltrations in hepatopancreatic tissue (grade 3) and (**B**) multifocal infiltration (grade 2) (black arrows). (**C**) Diffuse inflammatory infiltration in renal tubules (grade 3) and focal inflammatory process (grade 1) in (**D**).

Histological evaluation of inflammation (Figure 3) in *Diplodus sargus* from Sa observed a total of 30% of hepatopancreas, scored as follows: 10% (4/40) as grade, 1.15% (6/40) as grade 2, and 5% (2/40) as grade 3. In kidneys, inflammation was observed in 22.5% and scored as follows: 7.5% (3/40) as grade 1 and 15% (6/40) as grade 2. In *Diplodus sargus* samples from Na, inflammation was observed in 18.3% of hepatopancreas, scored as follows: 10% (6/60) as grade 3, 6.7% (4/60) as grade 2, and 1.7% (1/60) as grade 1. In kidneys, inflammation was observed in 25% and scored as follows: 16.7% (10/60) as grade 2, 5% (3/60) as grade 3, 3.3% (2/60) as grade 1, and 10% (6/60) of livers as grade 3. In *Sarpa salpa* from Sa, inflammation was observed in 11.1% (5/45) of hepatopancreas, scored as grade 2, and in 2.2% (1/45) of kidney scored as grade 2. In *Sarpa salpa* from Na, inflammation was observed in 11.2% of hepatopancreas, scored as follows: 5.6% (3/54) as grade 2, 3.7% (2/54) as grade 1, and 1.9% (1/54) as grade 3. Inflammation in the kidney resulted in 53.7%, scored as follows: 37% (20/54) as grade 1, 12.9% (7/54) as grade 2, and 3.7% (2/54) as grade 3.

### 3.3. Immunohistochemical Examination

**Figure 4:**
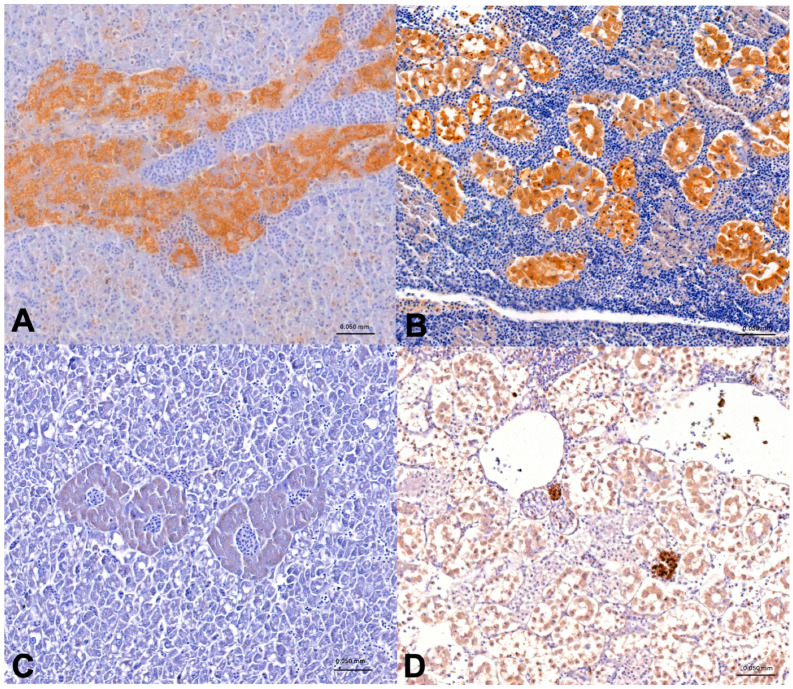
CYP1A1 immunohistochemistry in hepatopancreas (**A**,**C**) and kidney (**B**,**D**), (40×). Diffuse intracytoplasmic immunopositivity to CYP1A1 in hepatopancreas (**A**) and kidney (**B**) of *D. sargus* from Na. Focal intracytoplasmic immunopositivity to CYP1A1 in hepatopancreas (**C**) and kidney (**D**) of *S.salpa* from Sa.

Immunopositivity to CYP1A1 was observed in hepatopancreatic cells and renal tubular epithelial cells (Figure 4A–D). In particular, in *Diplodus sargus* from Na CYP1A1, immunopositivity was observed in 10% (6/60) of hepatopancreas with focal cytoplasmic immunopositivity, and 6.7% (4/60) of hepatopancreas and 1.7% of kidneys (1/60) with diffuse cytoplasmic immunopositivity. In the same species from Sa, 5% (2/40) of hepatopancreas showed focal cytoplasmic immunopositivity, and 12.5% (5/40) of hepatopancreas and 2.5% (1/40) of kidneys showed diffuse cytoplasmic immunopositivity. Moreover, in *Sarpa salpa* from Sa, 4.4% (2/45) of kidneys showed diffuse cytoplasmic, and 2.2% (1/45) of kidneys and hepatopancreas showed focal cytoplasmic immunopositivity. In the studied Na area, only 1.9% (1/54) of *Sarpa salpa* hepatopancreas showed focal cytoplasmic immunopositivity.

### 3.4. Statistical Analysis

The pathological changes showed a statistically significant difference in inflammation of the kidneys (*p* < 0.0001) between *S. salpa.* of both Gulfs. In addition, we found a statistically significant difference in the assessment of the increase in MMs/MMCs (*p* = 0.0384) and circulation disorders (*p* = 0.0325) of the hepatopancreas in *D. sargus* of both Gulfs. The other parameters examined showed no statistically significant differences.

## 4. Discussion

Due to the significance of the persistence and relevant effects of the exposure to PCBs [31], as well as heavy metals, it is of high importance to evaluate the contamination level among fish products that are frequently consumed in the human diet. In particular, the fish species under investigation belong to the Sparidae family, two sedentary species that show different trophic habits. Indeed, while *Sarpa salpa* is an obligate herbivore feeding mainly on Posidonia oceanica, *Displodus sargus* is an omnivore species that inhabits the rocky seabed, [25,30,32]. Therefore, these two species could be used as a valid tool for biomonitoring environmental pollution, in the context of different diets and ecosystem settings. To the best of the authors’ knowledge, this is the first study involving these species as pollution indicators through the observation of pathological lesions.

The concentrations of the six investigated PCB congeners were consistently found to be lower in the muscle than the legislative limits (Commission Regulation (EU) No 1259/2011), which established for fish muscle, fishery products, and derived products a limit value corresponding to 75 ng/g of wet weight, taking into consideration the measurement uncertainty and the dilution factor linked to sample pooling. Furthermore, from the comparison between the two organs and the two sampling areas, the concentration of PCBs was higher in the hepatopancreas of both species than in the muscle, as also reported in Diplodus s. by the study of [33]. In fact, this difference found its explanation because, normally, the liver is involved in high metabolic activity with consequent assimilation and accumulation [33]. Contaminants, such as heavy metals and PCBs, have a strong chemical affinity to lipid-rich tissues, such as liver and fat, where they accumulate [32]. This process is well known as bioaccumulation [22]. The concentration of a contaminant bioaccumulated in the food chain increases at the upper trophic levels, where it could induce a critical effect on human health [34]. This also explains the differences in PCB levels between the two selected species, where the concentration was higher in *Diplodus sargus* than in *Sarpa salpa*. In fact, according to another Italian study, *S. salpa* showed a lower concentration of PCBs compared to the fish T. trachurus, in which the concentration was about five times higher than the EC limit [35]. Herbivores, like *S. salpa*, have less fat content and consequently display less bioaccumulation than predatory fish or omnivores like *D. sargus*. [36]. The sea bream is a diurnal omnivore; it usually feeds on algae, sea urchins, worms, gastropods, and amphipods [37], while the salpa is strictly herbivorous in its sub-adult and adult stages; only the juveniles of this species feed on plankton [38]. By comparing the species, an opposite situation emerged concerning the organic contaminants, in which the *Sarpa salpa* presented a higher level of basal contamination than the *Diplodus sargus.* This difference between the lead levels per species was detected in the area of the Gulf of Naples, as well as in other geographical regions [33,36,39,40,41,42,43,44,45]. Comparing the two areas, PCBs concentration was higher in Salerno than the Napoli area for both species (49.3 and 51.0 ng/g of fresh weight for the hepatopancreas of *Diplodus sargus* and *Sarpa salpa*, respectively). This result was partially supported by the histological evaluation of circulatory disorders and increased number of MMs/MMCs in hepatopancreas of *D. sargus*. In fact, 37.5% of both organs showed circulatory disorders such as hemorrhage and congestion. The highest percentage of cases with an increased number of MMs was detected in both organs and in both species from Salerno (15% in *D. sargus* and 11,20% in hepatopancreas of *S. salpa*). Among the 15% of both organs of *D. sargus*, 7.5% (3/40) showed a grade 1; 5% (2/40) showed a grade 3; 2.5% (1/40) showed a grade 2 in the score system. These results were in accordance with chemical and statistical analysis, with significant differences in the assessment of the increase in MMs/MMCs (*p* = 0.0384) and circulation disorders (*p* = 0.0325) of the hepatopancreas in *D. sargus*. Thus, the increase in the number of melanomacrophages could be related to stress conditions in fish species, primarily pollutants [46]. These data do not confirm, with absolute certainty, that the increase in the number of MMs and circulatory disorders in *D. sargus* from the Salerno area is caused by the higher PCB values detected. Other stress factors must be considered, such as parasites, temperature changes, and other diseases that have not been considered in this study [47]. Another important element to consider is the inflammatory pattern. Kidneys of both species from the Na area showed a higher number of cases than Sa with inflammatory injury, 16,7% (10/60) in *Diplodus sargus* and 37% (20/54) in *Sarpa salpa*, respectively. We expected a significant expression of CYP1A1 in hepatopancreas and kidney samples from Salerno, especially in the *Diplodus sargus* specimen, especially considering the classification of the marine coast waters of the Campania Region, completed by the Regional Agency for Environmental Protection of Campania according to Ministerial Decree 260/10, from 2013 to 2018. In fact, the areas of Vico Equense (Punta Grandelle), Massa Lubrense (Punta Campanella), Amalfi, and the Gulf of Naples showed concentrations of lead, cadmium, and Σ (dioxins, furans, and PCBs) higher than the environmental quality standard. The chemical status of water bodies was considered not good. Although the expression of CYP1A1 was observed mostly in the hepatopancreas of *Diplodus sargus* from Sa (12.5%), compared to ones from the Na area (10%), there was no statistically significant difference. The substantial difference between hepatopancreas and kidneys may lie in the antioxidant defense role in fish of hepatopancreas, as it is already described in previous studies [48,49], but no specific data on fish are available on CYP1A1 expression in hepatopancreas.

From the histological analysis of gonadal stage maturation, *Diplodus sargus* individuals showed gonads in the emission phase in correspondence with the increase in temperatures and photoperiod, and parameters influencing sexual activity and deposition [50]. The long period in which *Diplodus sargus* with gonads in the gametic emission phase was observed indicated that the environmental conditions were favorable for incubation and larval development [51]. Hermaphroditism in *Diplodus sargus* was observed in March and May, contrary to what was reported in the literature [50], indicating a negative effect of the environment on gonadal differentiation. The *Sarpa salpa* individuals taken in both sampling areas showed a different gonadal development [52]. However, what was observed in the present study is in accordance with what is described in *Sarpa salpa* samples caught in the Canary Islands, where quiescent gonads were observed from January to March [52].

One of the limits of our study was the absence of gills analysis. In fact, as an indicator of water pollution, they could have given us more information on the levels of environmental contamination (14). This represents a preliminary study laying the foundations for future investigations on other species as well as predators at higher levels in the food chain.

## 5. Conclusions

The environmental quality and health status of a protected marine ecosystem constantly subjected to anthropogenic pressures is an endpoint that can be achieved by studying the pathological alterations and measuring pollutants in teleosts. The evaluation is very economical and easily achievable, but some variables related to the histological sign are not pollutant-based. As mentioned before, stressors such as temperature, starvation, parasites, and other diseases could change the state of these parameters [39]. However, based on the classification of the coastal marine waters of the Campania Region by the Regional Agency for Environmental Protection of Campania, we can state that the areas of Vico Equense (Punta Grandelle), Massa Lubrense (Punta Campanella), Amalfi, and the Gulf of Naples showed waters, based on pollutants found at concentrations above the standard, moderately polluted. This study could represent a starting point for future investigations and studies to fully correlate the role of pollutants to CYP1A1 in fish hepatopancreatic cells and to suggest insights into their role.

## Figures and Tables

**Table 1:** Lead (mg/kg) edible part *Sarpa s*.; LB = lower bound (<LOQ = 0 mg/kg).

Sample Number	Value
ID.1	0.032
ID.2	<0.020
ID.3	0.224
ID.4	0.03
ID.5	0.063
ID.6	<0.020
ID.7	0.022
ID.8	0.041
ID.9	0.034
ID.10	0.034
ID.13	0.052
ID.14	<0.020
ID.15	<0.020
ID.16	0.032
ID.17	0.046
ID.18	<0.020
ID.19	<0.020
ID.20	0.049
ID.21	<0.020
ID.22	<0.020
ID.23	0.171

**Table 2:** Lead (mg/kg) edible part *Diplodus s*.; LB = lower bound (<LOQ = 0 mg/kg).

Sample Number	Value
ID.8	<0.020
ID.9	<0.020
ID.10	<0.020
ID.11	0.035
ID.12	<0.020
ID.13	<0.020
ID.14	<0.020
ID.15	<0.020
ID.16	<0.020
ID.17	0.037

**Table 3:** Summary of the histopathological results for the different categories analyzed. Circulatory disturbances, increase in MMs, and MMC number and inflammation (%).

Species/Area	Organ	Circulatory Disturbances	MMs MMCs	Inflammation
*Diplodus s*. Na	Hepatopancreas	16.70%	1.70%	18.30%
	Kidney	21.70%	8.30%	25%
*Diplodus s*. Sa	Hepatopancreas	37.50%	15%	30%
	Kidney	37.50%	15%	22.50%
*Sarpa s*. Na	Hepatopancreas	11.10%	3.70%	11.20%
	Kidney	18.50%	0%	53.70%
*Sarpa s*. Sa	Hepatopancreas	22.20%	11.20%	11.10%
	Kidney	11.10%	6.70%	2.20%

**Table 4:** MMs and MMCs score system results.

Species/Area	Score	Hepatopancreas	%	Kidney	%
*Diplodus s*. Na	0	59	98.3	55	91.7
	1	1	1.7	1	1.7
	2	0	0	2	3.3
	3	0	0	2	3.3
*Diplodus s*. Sa	0	34	85	34	85
	1	3	7.5	3	7.5
	2	1	2.5	1	2.5
	3	2	5	2	5
*Sarpa s*. Na	0		96.3	54	100
	1	0	0	0	0
	2	2	3.7	0	0
	3	0	0	0	0
*Sarpa s*. Sa	0	40	88.9	42	93.4
	1	1	2.2	1	2.2
	2	3	6.7	2	4.4
	3	1	2.2	0	0

## Data Availability

All data used in the current study are available from the corresponding author upon reasonable request.
